# A CAD/CAM Zirconium Bar as a Bonded Mandibular Fixed Retainer: A Novel Approach with Two-Year Follow-Up

**DOI:** 10.1155/2017/1583403

**Published:** 2017-07-27

**Authors:** Maen Zreaqat, Rozita Hassan, Abdul Fatah Hanoun

**Affiliations:** Orthodontic Department, Universiti Sains Malaysia, 15160 Kubang Kerian, Kelantan, Malaysia

## Abstract

Stainless steel alloys containing 8% to 12% nickel and 17% to 22% chromium are generally used in orthodontic appliances. A major concern has been the performance of alloys in the environment in which they are intended to function in the oral cavity. Biodegradation and metal release increase the risk of hypersensitivity and cytotoxicity. This case report describes for the first time a CAD/CAM zirconium bar as a bonded mandibular fixed retainer with 2-year follow-up in a patient who is subjected to long-term treatment with fixed orthodontic appliance and suspected to have metal hypersensitivity as shown by the considerable increase of nickel and chromium concentrations in a sample of patient's unstimulated saliva. The CAD/CAM design included a 1.8 mm thickness bar on the lingual surface of lower teeth from canine to canine with occlusal rests on mesial side of first premolars. For better retention, a thin layer of feldspathic ceramic was added to the inner surface of the bar and cemented with two dual-cured cement types. The patient's complaint subsided 6 weeks after cementation. Clinical evaluation appeared to give good functional value where the marginal fit of digitized CAD/CAM design and glazed surface offered an enhanced approach of fixed retention.

## 1. Introduction

Stainless steel alloys containing nickel (8–12%) and chromium (17–22%) are widely utilized in the daily practice of orthodontics [1, 2]. Nickel improves the anticorrosive property of the alloy and decreases the ductility while chromium facilitates the formation of an anticorrosive passive film. These metals raise problems concerning their biocompatibility according to their performance in the oral cavity. The oral environment is particularly ideal for biodegradation of metals due to electrochemical breakdown driven by its ionic, thermal, microbiologic, and enzymatic properties [3, 4]. The saliva serves an excellent medium leading to release of metal ions. The potential health hazards from exposure to nickel and chromium have been scrutinized for long time. These include hypersensitivity, dermatitis, asthma, and cytotoxicity [5–7]. Changes in the oral mucosa and mutagenic potential are considered as well [8, 9].

Several studies have evaluated the salivary levels of nickel and chromium in biological fluids (in vitro) [10] and in the oral cavity (in vivo) [11, 12]. In all cases, concentrations did not reach toxic levels. However, metal tolerance and toxicity are not well understood. Thus, it can not be excluded that even nontoxic concentrations might produce cytotoxic changes. Moreover, the long-term exposure, as in orthodontic treatment, could limit the recovery time needed for cellular repair. Biocompatibility of orthodontic materials is a real concern as clinicians do not want to place orthodontic appliances with a risk of adverse toxic effects in their patients.

This case report describes for the first time a computer-aided design/computer-aided manufacturing (CAD/CAM) zirconium bar as a bonded mandibular fixed retainer with 2-year follow-up in a patient who was subjected to long-term treatment with fixed orthodontic appliance and suspected to have metal hypersensitivity as shown by the marked increase of nickel and chromium concentrations in a sample of the patient's unstimulated saliva.

## 2. Case Report

The case was of a 22-year-old woman complaining of headache for 18 months with severe episodes lasting around 6 hours, recurring about twice a month. Pain was located on the parietal regions, spreading to the orbit. Attacks were accompanied by nausea severe enough to hinder routine activities. The case was assessed by several specialists ruling out neurological, ophthalmological, and otorhinolaryngological pathologies. Cranial computed tomography (CT) and magnetic resonance imaging (MRI) revealed no pathology. Previous treatments considered nonsteroidal analgesics, physical therapy, hot and ice packs applications, and low-level laser therapy. All of them did not relieve pain to a satisfactory level. Her neurologist referred her to the orthodontic clinic suspecting a dilemma somewhere in her braces. The patient had fixed orthodontic appliance in the lower arch only with right first premolar extracted and space was closed with a satisfactory treatment outcome.

Clinical examination showed good oral hygiene with no signs of periodontal disease. Panoramic radiography (OPG) was normal. Occlusion was normal with no temporomandibular joint disorders. However, the patient reported that headache started 4 weeks after brackets bonding. At this stage, the patient was referred to a specialized center to test salivary biochemistry, particularly nickel and chromium levels. The chemical analyses were performed with a graphite furnace atomic absorption spectrometer using unstimulated salivary sample.

After consultation with patient's neurologist, a decision was made to remove braces as soon as possible and provide her with a CAD/CAM zirconium bar as a bonded mandibular fixed retainer as the patient refused to wear any removable appliance. The treatment objectives were to (1) remove braces and retain teeth at their present position and (2) fabricate a bonded mandibular fixed retainer being biocompatible in attempt to relieve the patient's complaint.

## 3. Treatment Procedures

**Figure 1:**
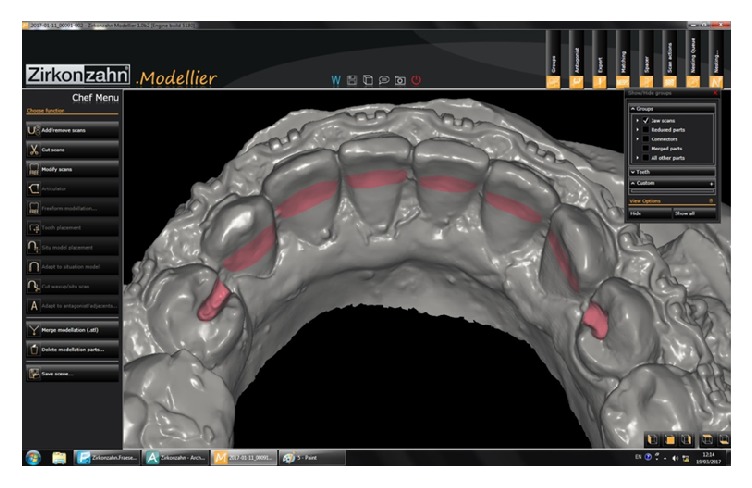
CAD/CAM zirconium bar survey.

**Figure 2:**
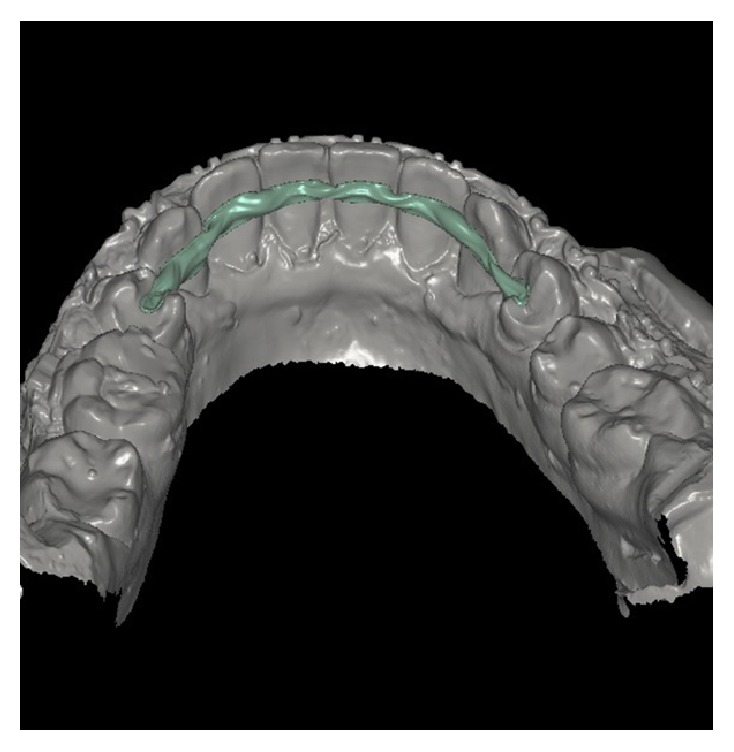
Virtual CAD/CAM zirconium bar design.

Occlusal rests of 2 mm depth were prepared on the mesial surface of first premolars. Brackets were covered with wax prior to impression taking to facilitate its removal. A definitive impression of the mandibular teeth was made with an alginate impression material and the master model was prepared from type IV stone (ResinRock, Whip Mix Europe, Dortmund, Germany). The working cast was mounted on articulator and scanned in the S600 ARTI scanner (Ice Zirkon, Zirkonzahn-ZA9246A, Italy) to transfer the baseline landmarks from the mandibular arch directly to the software (Figure 1). The CAD/CAM design included a 1.8 mm width and a 1.4 mm thickness zirconium bar extended on the lingual surface of lower teeth from canine to canine and at 3.5 mm away from the free gingival margin. Occlusal rests were connected to the bar as well (Figure 2). The finished design was directly milled using Zirkonzahn's Screw-Tec system.

**Figure 3:**
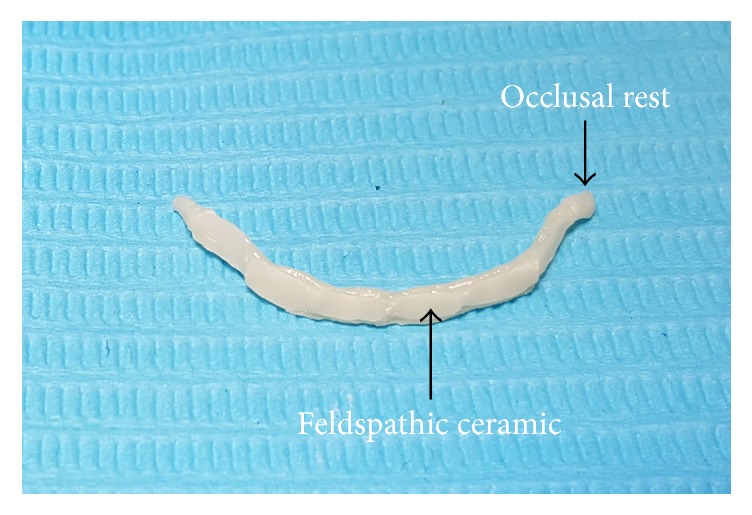
CAD/CAM zirconium bar prior to cementation.

**Figure 4:**
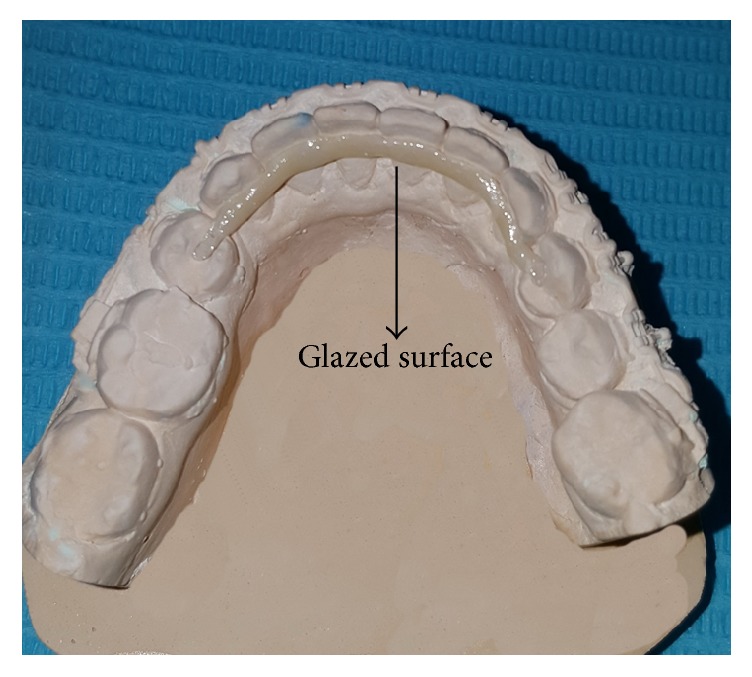
CAD/CAM zirconium bar fitted into the cast.

Zirconium does not chemically adhere to enamel; therefore, the inner surface of the milled bar facing teeth was reduced manually and placed under an infrared lamp to dry for 40 minutes and then sintered overnight at 1,600°C in a sintering furnace Zirkonofen 700. Later, a thin layer of feldspathic ceramic was fired onto the bar (Figure 3) and subsequently received the treatments sandblast, hydrofluoric acid etching, and silane coupling while the outer part was glazed (Figure 4). The try of insertion of the entire bar into the patient's mouth proceeded properly ensuring good marginal fitness. The retainer was cemented with two dual-cured cement types (Variolink II and RelyX ARC). The occlusal rest on the left side needed minimal reduction (Figure 4).

## 4. Treatment Results

Salivary spectrophotometry analysis showed that nickel concentration was 4.230 *μ*g/L and chromium was 12.520 *μ*g/L. These concentrations were much lower than estimated toxic levels but comparable to the average dietary intake of nickel (200–300 *μ*g/day) and chromium (280 *μ*g/day) [13]. Several in vitro studies found that release reached a maximum within the first month of boding and then diminished due to formation of surface oxide film which resists corrosion, thus slowing down the release of metals [14]. However, the case is different here where measurable amounts of nickel and chromium release were recorded after 22 months of treatment alerting possible association with the complaint.

**Figure 5:**
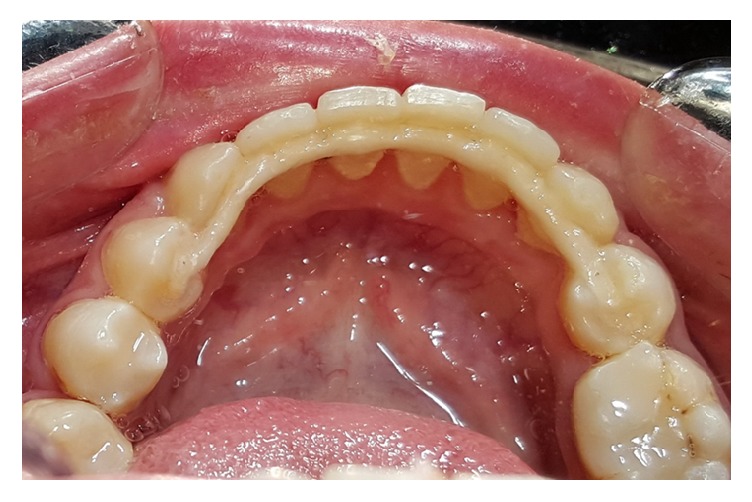
Bonded fixed retainer in situ, 2-year follow-up.

The patient reported pain relief two weeks following cementation, and headache subsided completely after 6 weeks. Another atomic absorption spectrometer analysis was performed to test salivary nickel and chromium levels which showed negligible concentrations which might explain the patient's complaint. The patient was reviewed regularly for two years reporting no complaint (Figure 5). Clinical evaluation appeared to give good functional value where the marginal fit of digitized CAD/CAM design and glazed surface offered an enhanced approach of fixed retention.

## 5. Discussion

Retention is the phase of orthodontic treatment which maintains teeth in their orthodontically corrected positions, following the cessation of an active orthodontic tooth movement. Retention after active orthodontic treatment is essential for preventing relapse as the posttreatment stability of any corrected malocclusion is unpredictable. Removable retainers such as Hawley retainers and clear overlay retainers have been typically used in the retention phase [15]. On the other hand, fixed retainers are indicated for long-term retention of the labial segments particularly in the lower arch. Over time, there was a definite shift in preferred retainer types from removable to fixed retainers due to their durability and the less patient's compliance needed [16]. This case report describes the use of a CAD/CAM zirconium bar as a bonded mandibular fixed retainer. The advantage of CAD/CAM technology includes the ease and accuracy of replicating details such as guiding planes, rest seats, and retentive undercuts providing high marginal integrity with minimal adjustments. Zirconia ceramics are prime candidates in oral rehabilitation due to their high mechanical strength, flexural resistance, and long-term biocompatibility [17, 18]. The mechanical properties of zirconia ceramics are considerably higher than other dental ceramics with their flexural strength of 900–1200 MPa, fracture toughness of 9-10 MPa, compressive strength of 2000 MPa, and young modulus of 210 GPa [19–21]. Although their brittleness remains a concern, zirconium bar in this case is shielded by being away from the heavily loaded functional areas which reduces the risk of chipping considerably [22].

The oral environment is considered hostile and potentially corrosive and metal brackets are important cellular stress factor. Metal toxicity is a common finding in studies related to the biocompatibility of orthodontic materials. Unfortunately, the cellular and molecular mechanisms responsible for this predilection are still unclear. Future work in more clinically relevant situations will lead to a better understanding of the clinical effects of corrosion. Moreover, alterations in the CAD/CAM design may consider its bonding to the lingual surface of lower canines only enhancing a sound periodontal status.

Occlusal preparation of bicuspids might be iatrogenic. Frictional heat and vibrations generated by high speed burs during teeth preparation may jeopardize the biologic entity of dentin-pulp complex. However, minimal removal of dental structure was performed with a good quality of marginal finish lines.

## 6. Conclusion

In case of history of nickel and chromium sensitivity, the technique could be considered as a novel method to fabricate retrofitted surveyed retainers. With their excellent biocompatibility and high mechanical properties, zirconia ceramics are preferable alternative in oral rehabilitations.

## References

[1] Kerosuo H., Moe G., Hensten-Pettersen A. (1997). Salivary nickel and chromium in subjects with different types of fixed orthodontic appliances. *American Journal of Orthodontics and Dentofacial Orthopedics*.

[2] Hwang C.-J., Shin J.-S., Cha J.-Y. (2001). Metal release from simulated fixed orthodontic appliances. *American Journal of Orthodontics and Dentofacial Orthopedics*.

[3] Barrett R. D., Bishara S. E., Quinn J. K. (1993). Biodegradation of orthodontic appliances. Part I. Biodegradation of nickel and chromium in vitro. *American Journal of Orthodontics and Dentofacial Orthopedics*.

[4] Eliades T., Trapalis C., Eliades G., Katsavrias E. (2003). Salivary metal levels of orthodontic patients: A novel methodological and analytical approach. *European Journal of Orthodontics*.

[5] Burrows D. (1986). Hypersensitivity to mercury, nickel and chromium in relation to dental materials. *International Dental Journal*.

[6] Fisher J. R., Rosenblum G. A., Thomson B. D. (1982). Asthma Induced by Nickel. *The Journal of the American Medical Association*.

[7] Romaguera C., Grimalt F., Vilaplana J. (1988). Contact dermatitis from nickel: an investigation of its sources. *Contact Dermatitis*.

[8] Natarajan M., Padmanabhan S., Chitharanjan A., Narasimhan M. (2011). Evaluation of the genotoxic effects of fixed appliances on oral mucosal cells and the relationship to nickel and chromium concentrations: An in-vivo study. *American Journal of Orthodontics and Dentofacial Orthopedics*.

[9] Costa M., Mollenhauer H. H. (1980). Carcinogenic activity of particulate nickel compounds is proportional to their cellular uptake. *Science*.

[10] Retamoso L. B., Luz T. B., Marinowic D. R. (2012). Cytotoxicity of esthetic, metallic, and nickel-free orthodontic brackets: Cellular behavior and viability. *American Journal of Orthodontics and Dentofacial Orthopedics*.

[11] Sahoo N., Kailasam V., Padmanabhan S., Chitharanjan A. B. (2011). In-vivo evaluation of salivary nickel and chromium levels in conventional and self-ligating brackets. *American Journal of Orthodontics and Dentofacial Orthopedics*.

[12] Hafez H. S., Selim E. M. N., Kamel Eid F. H., Tawfik W. A., Al-Ashkar E. A., Mostafa Y. A. (2011). Cytotoxicity, genotoxicity, and metal release in patients with fixed orthodontic appliances: A longitudinal in-vivo study. *American Journal of Orthodontics and Dentofacial Orthopedics*.

[13] Bhaskar V., Subba Reddy V. (2010). Biodegradation of nickel and chromium from space maintainers: An in vitro study. *Journal of Indian Society of Pedodontics and Preventive Dentistry*.

[14] Martín-Cameán A., Jos Á., Mellado-García P., Iglesias-Linares A., Solano E., Cameán A. M. (2015). In vitro and in vivo evidence of the cytotoxic and genotoxic effects of metal ions released by orthodontic appliances: A review. *Environmental Toxicology and Pharmacology*.

[15] Keim R., Gottlieb E., Nelson A., Vogels D. (2002). Study of orthodontic diagnosis and treatment. Part 1. Results and trends. *Journal of Clinical Orthodontics*.

[16] Pratt M. C., Kluemper G. T., Hartsfield J. K., Fardo D., Nash D. A. (2011). Evaluation of retention protocols among members of the American Association of Orthodontists in the United States. *American Journal of Orthodontics and Dentofacial Orthopedics*.

[17] Alghazzawi T. F. (2016). Advancements in CAD/CAM technology: Options for practical implementation. *Journal of Prosthodontic Research*.

[18] Gautam C., Joyner J., Gautam A., Rao J., Vajtai R. (2016). Zirconia based dental ceramics: structure, mechanical properties, biocompatibility and applications. *Dalton Transactions*.

[19] Elsaka S. E., Elnaghy A. M. (2016). Mechanical properties of zirconia reinforced lithium silicate glass-ceramic. *Dental Materials*.

[20] Komine F., Blatz M. B., Matsumura H. (2010). Current status of zirconia-based fixed restorations. *Journal of oral science*.

[21] Manicone P. F., Rossi Iommetti P., Raffaelli L. (2007). An overview of zirconia ceramics: basic properties and clinical applications. *Journal of Dentistry*.

[22] Ozkurt Z., Kazazoglu E. (2010). Clinical success of zirconia in dental applications. *Journal of Prosthodontics*.

